# Sex differences and impact of body mass on performance from childhood to senior athletes in Olympic weightlifting

**DOI:** 10.1371/journal.pone.0238369

**Published:** 2020-09-03

**Authors:** Marianne Huebner, Aris Perperoglou

**Affiliations:** 1 Department of Statistics and Probability, Michigan State University, East Lansing, MI, United States of America; 2 School of Mathematics, Statistics and Physics, Newcastle University, United Kingdom; São Paulo State University (UNESP), BRAZIL

## Abstract

**Background:**

Olympic weightlifting requires technical skills, explosive power, strength, and coordination. Weightlifters can be competitive within a range of morphological characteristics due to competition body weight classes. To date no studies have examined when sex differences arise in weightlifting and the impact of body mass on performances at different ages.

**Objectives:**

To examine when sex-related differences emerge, to quantify the influence of body mass on performances at different ages, and to estimate the age at peak performance.

**Methods:**

Competitions results from USA Weightlifting National Championships, Youth, Junior, and Senior from 2014 to 2019 were collected for weightlifters aged 6 to 30.

**Results:**

At age 10 the median total weight lifted was 51kg and 54kg, respectively, for girls and boys. From age 10 to 12 a gender gap emerges with a sex difference of 11.7% at age 14 at 55kg body mass. At age 25 the sex-related performance difference is smaller for lighter athletes (23.6% at 69kg body mass) and larger for heavier athletes (29.9% at 81kg body mass). The median peak age for men is 26.5 years (95% CI: 25.7, 27.3) and for women 25.9 years (95% CI: 24.7, 27.3).

**Conclusion:**

We quantified the impact of body weight and age and sex differences for youth and young adults, ages 6 to 30 years old, participating in national level Olympic weightlifting competitions in the United States. Body weight at younger ages has less impact on performance compared to older ages, and boys and girls perform similarly. When reaching the ages typically associated with the onset of puberty, boys’ performances rapidly increase and the gap between genders widens. Women achieve peak performance at a similar age than men. Such results may help to establish progression trajectories for talented athletes and inform coaches, athletes and national governing bodies.

## Introduction

Competitive sports performance increases during adolescence and has been studied in sports such as track and field and swimming in different countries [1–4]. Differences in the performance development in these types of sport and Olympic weightlifting arise due to a training emphasis on speed, endurance, strength, or propulsive power. In weightlifting athletes lift the weight from the floor to overhead. The vertical velocity of the barbell can reach up to 2.28 and 1.73 and m/sec in snatch and clean & jerk, respectively [5,6]. Weightlifting exercises require technical skills, power, strength, speed, balance, and coordination [6,7] and have become popular to augment training of athletes in other sports. Weightlifting training leads to an adaptation of musculoskeletal and cardiovascular systems, leading to greater cross-sectional areas of type-II fibers and lean tissue in limbs, and the power output of weightlifters exceeds that of other strength athletes [5, and references therein]. To date no studies addressed when sex differences arise in weightlifting and what impact body mass has on performances at different ages.

Youth development has been studied for ages 11 and up in track and field disciplines [1–3,8] but younger children have only been examined in swimming and running [4,8]. In these sports sex related differences in athletic performance emerge at around 12 years of age and reach adult plateau in the late teenage years [8]. Track and field is comprised of 21 disciplines and elite athletes within the specific disciplines likely have similar physique [9]. Weightlifting athletes compete in different body weight classes and thus can be competitive within a range of morphological characteristics. This enables us to study the effect of body mass on performance and peak age for male and female athletes in specific weightlifting exercises requiring explosive strength, and to compare such results with what has been found in jumping disciplines.

Studies of performance decline in sports have focused on Masters athletes, ages 35 and up [10–13]. However, the age at peak performance is before age 30 in some sports. The peak age varies by discipline in track and field where throwers have higher body mass and achieve peak performance at a later age than jumpers or runners [9,14,15]. Furthermore, differences in peak age were observed in different countries. Specifically for jumping disciplines, peak age was about 21 years for German and Italian athletes [1,2] and about 26 years for athletes globally [14,15]. Weightlifters from Western European countries at world championships peaked at younger ages than in other countries [16]. It is unlikely that such differences are solely due to biological reasons as participation requires financial resources and is affected by socioeconomic factors [17]. USA Weightlifting has recorded competition results from national youth, junior, and senior championships thus provides a large data set from the same country with a diverse population. The choice of data from national level competitions in the USA ensures that athletes’ technique is better developed than at regional competitions as they must meet a qualifying standard, and the quality of refereeing is more consistent. Youth championships include the age category 13 and under which is not available at world championships. Weightlifting has seen a tremendous growth in recent years due to the popularity of CrossFit, and the proportion of females has increased from 41% in 2014 to 52% in 2019 in these national championships. Female participation is still in its infancy in other countries. Thus a better understanding of performance development and sex-related differences based on this large national database may inform coaches, athletes, and national governing bodies.

The objective of this study was to quantify performance development in Olympic weightlifting and study the effect of body mass and sex on total weight lifted across ages 6 to 30 in a large national database. We hypothesized that in weightlifting 1. sex differences emerge during ages associated with the onset of puberty similar to other sports and vary by body mass, 2. body mass has less effect on performance in youth than in senior age categories, and 3. women and men peak at about the same age in their mid-twenties.

## Methods

### Study population

Competitions results from USA Youth National (up to 17 years), Junior National (ages 15 to 20), and Senior National Championships (15 and older) were obtained from USA Weightlifting (https://www.teamusa.org/usa-weightlifting/resources/results-archive). This included results from 2014 to 2019. Since athletes may participate in multiple competitions each year, only the highest performance per year for individuals was included. Since the performance declines in the late 20’s and participation after age 30 is sparse, only weightlifters until age 30 were included in the study.

**Fig 1:**
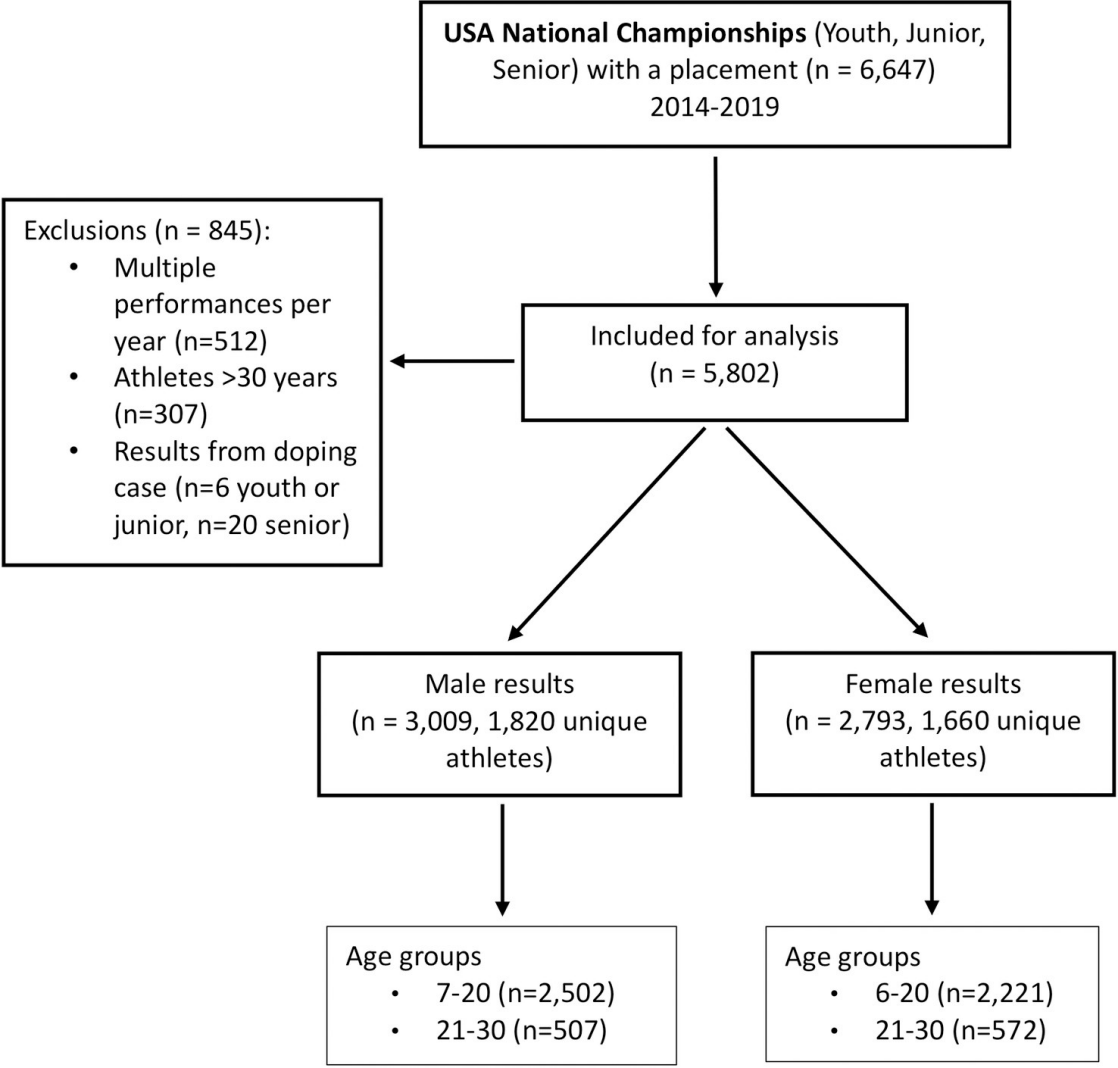
STROBE flow diagram.

In Olympic weightlifting competitions athletes lift the weight from the floor to overhead in a continuous movement (snatch), or lifting the weight to the shoulders and then overhead (clean and jerk). The total weight lifted is the sum of the best snatch weight and the best clean and jerk weight, if there was at least one valid attempt among three attempts in each of these lifts. The lifts are judged by three referees according to the same rules at all ages. Performance was defined as the total weight (kg) lifted. Exact body weights and ages were available for each competition result. All results from weightlifters who received a sanction due to doping offences were removed. Sanctioned athletes are listed on the website of USA Weightlifting (USAW) and the International Weightlifting Federation (IWF). Exclusions are described in Fig 1.

Ethics approval for this research was obtained from the Internal Review Board at Michigan State University and was granted an exempt status, since the data are publicly available.

### Statistical analysis

Continuous variables were summarized as median and 95% bootstrap confidence intervals or range. Wilcoxon rank sum tests were used for group comparisons. The sex difference for total weight lifted in kilograms was calculated as 100*(male total–female total)/male total.

Quantile regression models [18] for the total as a quadratic function of age were fitted with bootstrap sampling to estimate a confidence interval for age at peak performance. The age at peak performance is estimated by estimating the regression coefficients (a, b, c) of the model Total [kg] = a* Age + b* Age^2^ + c, and is given by -b/(2*a). Data for model fitting include the exact age at the first day of the competition rather than the competition age which is the age as of December 31 of the year of the competition. The median age at peak performance between 20 and 30 was estimated with a 95% bootstrap confidence intervals based on 500 bootstrap samples with 2000 resamples.

Due to variable training factors and qualifying standards for competitions at different age groups, as well as variation in the onset of puberty, we estimated performance levels by quantiles in this cross-sectional study. Quantile foliation is an extension of quantile regression and quantile sheets [19] and allows for estimating curves for median or other quantiles of performance across age and body mass. The leaves of the foliation are based on asymmetrically weighted least squares regression but with an extra smoothing dimension to prevent curves at different quantiles from crossing. In order to smooth the data across three dimensions, quantiles, age, and body mass, P-splines are utilized to ensure a smooth function along the axes [20].

All analyses were performed using the statistical software R v. 3.6.1 (2019) and the package gamlss v.5.1.4.

## Results

### Study population

A total of 5,802 performance results for male and female weightlifters aged 6 to 30 were used in the analyses (Fig 1). Children and adolescents up to age 17 accounted for 64.5% (n = 3,743). There were 48.1% (n = 2,793) female and 51.9% (n = 3,009) male performances from USA National Championships from 2014 to 2019. At ages below 16 approximately half are female, between ages 16 and 20 the proportion of female athletes is below that of male athletes with (40–45%) but increases in the senior class (53%) (Table 1). The number of participants at the National senior championships accounted for 19.8% (n = 1142) of the athletes, and 5.8% (n = 67) were under age 21 at this level.

**Table 1:** Number of competition results stratified by sex and age. Youth competition age categories are below 18, Juniors are ages 15–20, and Seniors ages 15 and older.

Age groups	Male (n = 3009)	Female (n = 2793)
13 and under	679 (50.3%)	672 (49.7%)
14–15	487(49.4%)	498 (50.6%)
16–17	751(53.4%)	656 (46.6%)
18–20	585 (59.7%)	395 (40.3%)
21–30	507 (47.0%)	572 (53.0%)

### Body mass and age of weightlifters

**Fig 2:**
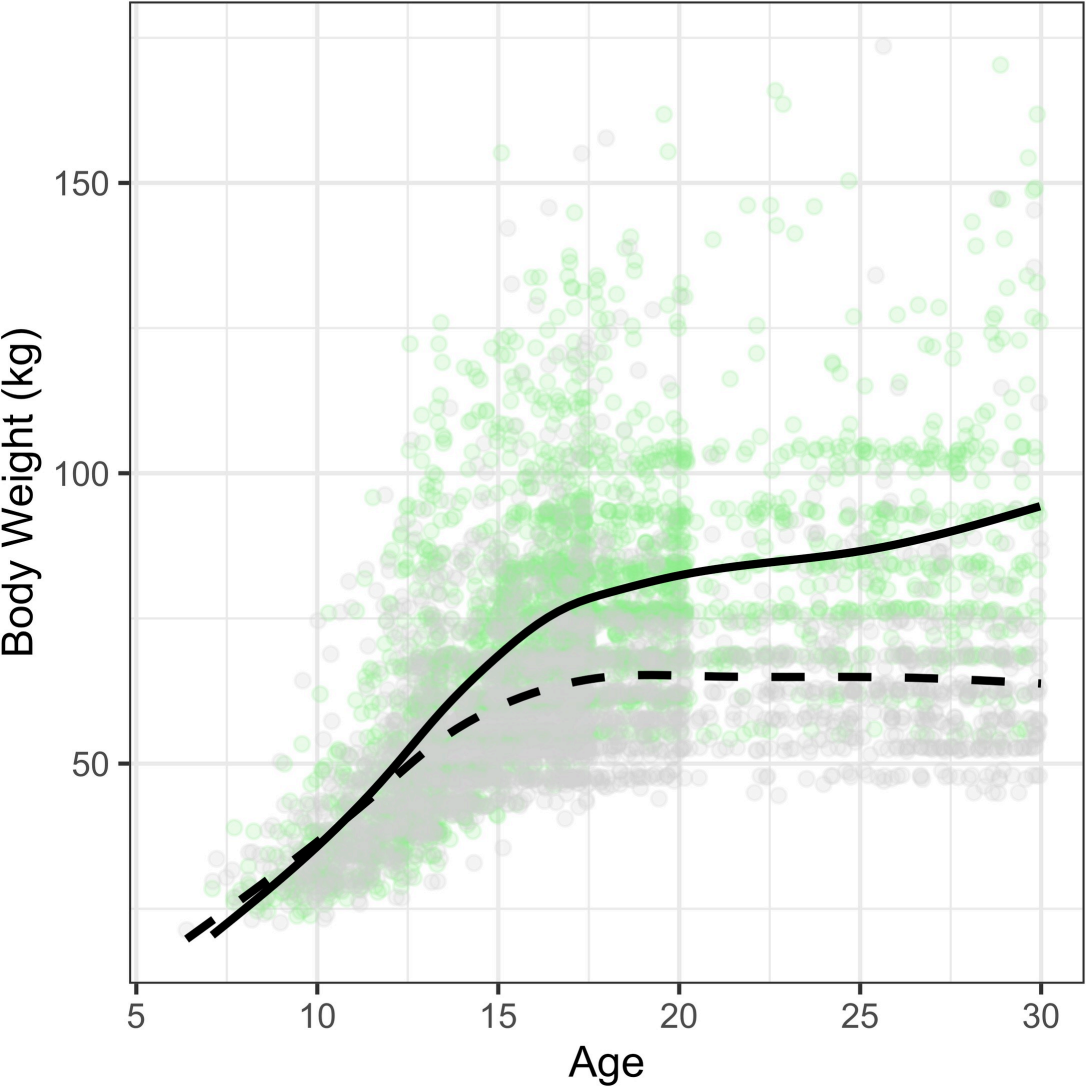
Individual data of age by body weight for males (green) and females (grey) overlaid with locally estimated regression curves.

As they grow older young weightlifters move into higher body weight classes. Up to age 13 males and females do not differ in body mass (Table 2, Fig 2), and the distribution is mostly unimodal with a few spikes that indicate body weight classes.

**Table 2:** Median (range) of body mass in kilograms for each age interval.

Age groups	Male (n = 3013)	Female (n = 2795)	p-value
6–9	29.0 (23.8–50.0)	30.5 (21.3–50.4)	0.191
10–11	35.4 (23.8–81.9)	35.9 (23.3–83.4)	0.915
12–13	49.5 (27.9–125.9)	49.0 (26.9–105.7)	0.063
14–15	63.8 (40.0–155.2)	56.7 (32.9–142.2)	<0.001
16–17	73.1 (46.3–144.9)	59.5 (40.5–155.1)	<0.001
18–20	76.6 (53.3–161.8)	62.3 (44.0–157.7)	<0.001
21–24	83.9 (54.3–165.9)	62.5 (44.5–102.9)	<0.001
25–27	84.5 (54.8–150.4)	62.6 (47.0–173.6)	<0.001
28–30	85.6 (54.2–170.3)	61.7 (44.9–147.3)	<0.001

**Fig 3:**
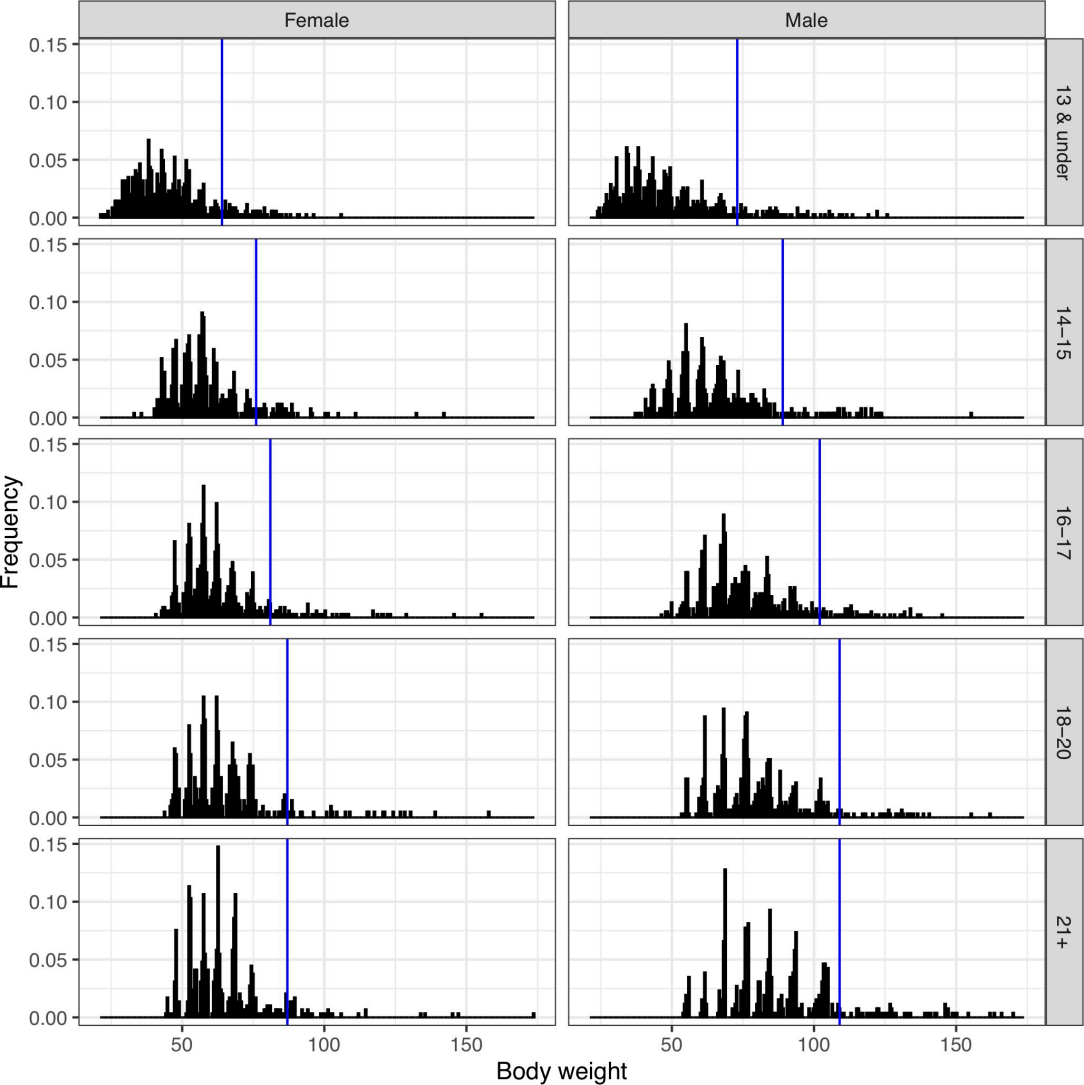
Histogram of body weights in different age groups. Lines indicate the unlimited upper body weight class.

The median body mass changed from 58 kg at age 14 to 76 kg at age 21 for males, and 53 kg to 62 kg for females (Table 2). For youth and adults, the body mass distribution is multi-modal with distinct spikes, since athletes strive to attain the maximum body weight possible within each competition body weight category (Fig 3).

Body weight categories are approved by the Executive Board of the IWF taking into account input from different countries. There is one unlimited body weight class in each age category where athletes gain more body mass in order to maximize the total weight lifted. The proportion of athletes in the heaviest IWF weight categories is largest for 13 and under, 17.2% boys and 10.7% girls and smallest for the junior age category, 18–20 years with 5 and 7%, respectively, for male and female athletes (Table 3).

**Table 3:** Number of athletes in the unlimited body weight categories (IWF/USAW body weight classes for age categories as of 2018).

Age group (male/female)	Men n (%)	Women n (%)
U13 (73/64 kg)	70 (17.2%)	72 (10.7%)
14–15 (89/76 kg)	45 (9.2%)	46 (9.2%)
16–17 (102/81 kg)	57 (7.2%)	53 (8.1%)
18–20 (109/87 kg)	30 (5.1%)	27 (6.8%)
21–30 (109/87 kg)	51 (10.1%)	41 (7.2%)

### Age-associated influence of body mass on performance

**Fig 4:**
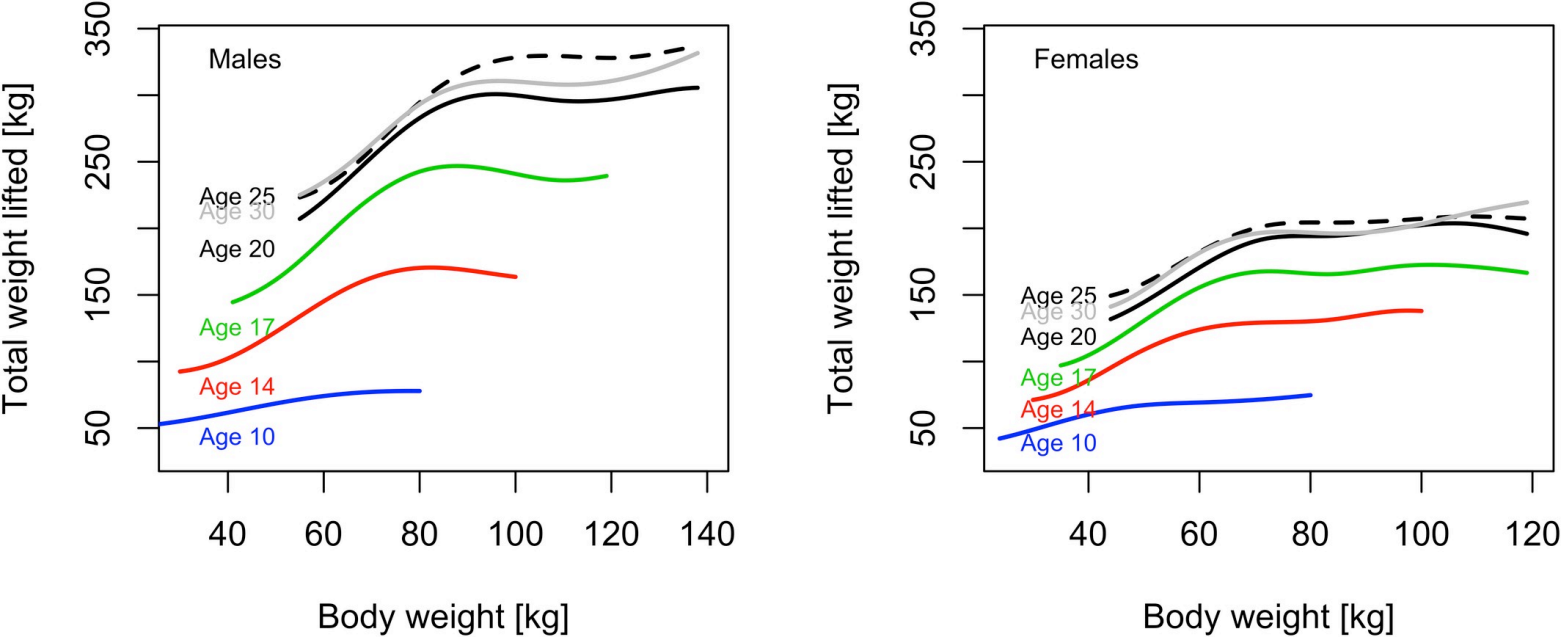
Male and female performance for different ages across bodyweight.

Athletes with higher body mass are generally able to lift more weight across all ages. However, body weight has a smaller effect on performance at younger ages in both male and female athletes. At age 10 there is 2 to 4% performance difference from lighter to heavier body mass. There is a steeper increase in total weight lifted with increasing body mass at older ages. The curves level off at the highest body mass. At age 30 the median performance is lower than at age 25 (Fig 4).

### Sex differences in performance

At age 10 the median total weight lifted is 54 and 51 kg, respectively, for boys and girls (p = 0.127), and at age 12 the median increased to 75 and 70 kg (p = 0.091), after which the gender gap widens and there is a steep increase in weight lifted, especially for males. Below age 10 girls perform better, or not worse, than boys at 35kg body mass, the median body mass at that age.

**Fig 5:**
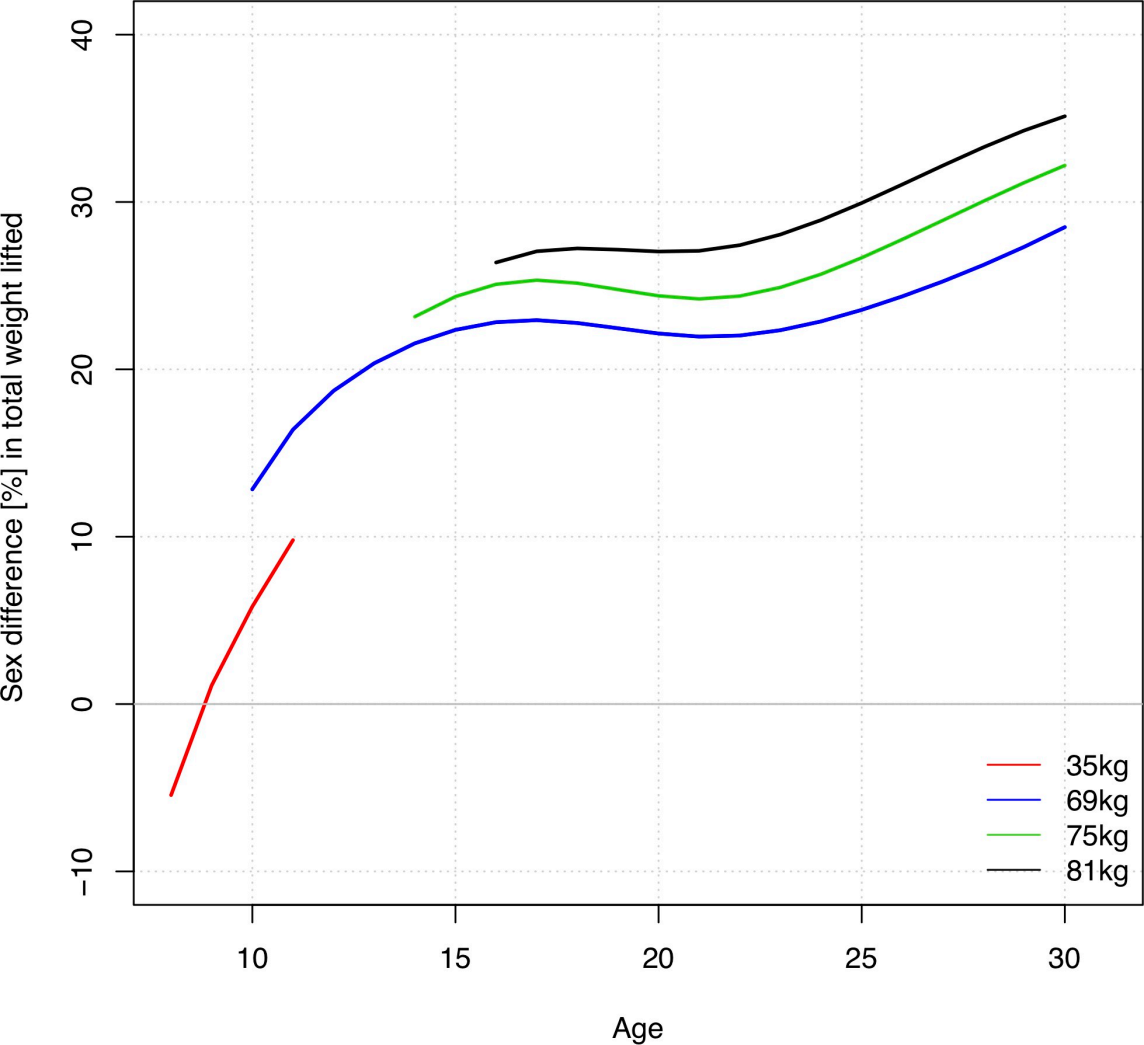
Sex differences in percent in total weight lifted for different body mass and ages at the 90^th^ percentile.

With older age the body mass increases. For lighter weightlifters at 55kg body mass the sex-related performance differences increases from 8.5% at age 10 to 19.5% and 23.6% ate ages 17 and 25, respectively, at the 90^th^ percentile. At 69kg body mass the sex-related performance difference increases from 12.8% at age 10 to 22.9% at age 17. At age 25 men outperform women by 23.6% at 69kg body mass and by 29.9% at 81kg body mass (Fig 5).

### Age at peak performance

Women reach the peak performance at a younger age than men at the 90^th^ percentile (p<0.001). The age at peak performance for men at the 90^th^ percentile is 26.5 (95% CI: 25.7, 27.3) years and for women 25.9 (95% CI: 24.7, 27.3) years. At the 50^th^ percentile the peak ages are 27.8 (95% CI: 27.1, 28.5) years for men and 27.6 (95% CI: 26.8, 28.4) years for women (Table 4).

**Table 4:** Age at peak performance for USA athletes and athletes at IWF world championships and Olympics* at the 90^th^ and 50^th^ percentile.

Percentile	Men peak age Median (95% CI)		Women peak age Median (95% CI)	
	USA	IWF	USA	IWF
90^th^	26.5 (25.7, 27.3)	25.6 (25.7, 27.1)	25.9 (24.7, 27.3)	25.7 (24.7, 27.5)
50^th^	27.8 (27.1, 28.5)	26.3 (25.6, 27.3)	27.6 (26.8, 28.4)	25.3 (24.7, 26.0)

*Reference: [16]

## Discussion

A total of 5,802 performances at USA National championships for weightlifters ages 6 to 30 years were analyzed. We studied the effect of body mass on performance, identified the timing and size of emerging sex-related differences in weightlifting performance from childhood to adolescence, and estimated performance trajectories from childhood to seniors at different performance levels for male and female athletes.

### Weightlifters have higher body mass than non-athletes

As they grow older young weightlifters move into higher competition body weight classes. Athletes with higher body mass are generally able to lift more weights in both sexes. Up to age 13 males and females did not differ in body mass, and the distribution was mostly unimodal with a few spikes that indicate weight classes. For youth and adults, the body mass distribution was multi-modal with distinct spikes, since athletes or their coaches strive to attain the maximum body weight possible within each competition body weight category to optimize performance. There is one unlimited body weight class in each age category where athletes gain more body mass in order to maximize the total weight lifted. A larger proportion of youth weightlifters can be expected to be heavier than controls of the same age in the general population. For example, for 14 to 20 year olds 33% male and 17% female weightlifters were heavier than the 90^th^ percentile on the Center for Disease Control (CDC) weight-for-age growth chart (https://www.cdc.gov/growthcharts/), and 13% male and 7% female weightlifters are heavier than the corresponding 97^th^ percentile for their age. However, the perception that weightlifters are heavier compared to athletes in other types of sport may only apply to the unlimited body weight category, which is more than 109kg and 87kg for men and women, respectively. In this study male weightlifters ages 18 to 21 had a mean body mass 80.0 ± 15.4 kg and females 63.7 ± 11.7 kg. The combined mean body mass for weightlifters in this age range was 72.5 ± 17.5 kg. In track and field there are no body weight classes. The comparison to jumping and throwing disciplines is relevant since these types of sport as well as weightlifting emphasize explosive strength. In throwing disciplines higher body mass is a prerequisite for success, but, due to the weight categories, weightlifters with lighter body mass can excel in competitions. For example, collegiate track and field athletes with a mean age 19.2 had a mean body mass of 78.4 ± 11.6 kg for men and 67.0 ± 14.2 kg for women. More specifically, sprinters were at 67.1 ± 8.8 kg, jumpers at 67.6 ± 9.1 kg, and throwers had the highest body mass for men and women overall 90.4 ± 18.3 kg [9]. The overall mean body mass for weightlifters was closer to the body mass of jumpers than that of throwers.

### Higher body mass may not lead to better performance at younger ages

While higher body mass is favorable for lifting more weight, it has a smaller effect on performance at younger ages. At age 10 body mass had little influence on the performance (2–4%). The increase in total weight lifted with increasing body mass was less steep for adolescents than for seniors. Furthermore, performance plateaued at higher body mass at all ages. Weightlifting requires force to move one’s own body as well as the weight, thus any performance advantage due to a heavier body mass is balanced by the additional strength it takes to move the body. Factors such as lean muscle mass in male and female weightlifters influence performance across all age and weight categories [5].

### Sex-related performance differs by age and body mass

With the rise of circulating testosterone in male puberty and the onset of gender divergence in fat-free muscle mass [21], there is a rapid increase in weightlifting performance during adolescence. The performance progression was more moderate for female than for male weightlifters during adolescence until reaching peak performance in their mid-twenties. Variation in performance among youth athletes at the same chronological age is to be expected due to the difference in timing and rate of biological maturation. The hormonal life cycle is also relevant for Masters athletes, ages 35 and up, where the performance decline was at first similar between men and women and then rapidly decreased for women during the transition to menopause as seen in weightlifting [10] as well as in high jump and throwing disciplines in track and field [11, Fig 2]. The sex differences for Masters were smaller in running and long jump [11], or in swimming [22,23].

Before puberty there were small sex-related differences in strength and jumping ability [3,8,24]. In a study on USA swimming, it was noted that girls are faster than boys prior to puberty [4]. In weightlifting, girls’ and boys’ performances were similar before age 10. After age 10 males outperformed females, and the difference was greater for athletes with higher body mass. At age 17 the gender gap in performance was 22.9% and increased to 23.6% at age 25 at 69kg body mass. For heavier athletes, at 81kg body mass, the gender gap was 29.9% at age 25. This was comparable to the gender gap for world records with 23.1% for ages 22 to 27 in the 69kg weight category, which was the only competition body weight category in common for men and women prior to 2019 (https://www.iwf.net/ Accessed May 7, 2020). In world records before 2009 there was a significant larger gender gap of 36.8% [25]. The growth of the participation rate of women likely contributed to the narrowing of the gender gap. The gender gap for weightlifters with lower body mass corresponded to that in jumping disciplines in track and field, 5.8% prior to puberty and 19.4% by age 17 [26]. For Masters age classes from 35 to 59 the difference between male and female USA weightlifting records ranges from 21.7 to 48% (http://mastersweightlifting.org/. Accessed May 7, 2020). Elite athletes may exit competitive-level weightlifting when their performance starts declining or may continue with less training intensity at recreational levels which would explain the wider gender gap in Masters age classes [16,17].

### Women and men have similar age at peak performance

Weightlifting performance increased for adolescents at first rapidly, then at a slower rate until it reached a plateau in the mid-twenties, after which the performance started declining. The advantage of using a quantile smoothing approach is that it allows identification of different patterns at different performance levels as measured by quantiles. The rate of improvement was faster for adolescents at higher quantiles compared to lower quantiles, and peak ages differed. Such performance trajectories may be relevant for identifying talented athletes. The age at peak performance for men at the 90^th^ percentile was 26.5 years (CI: 25.7, 27.3) and for women 25.9 years (CI: 24.7, 27.3) at USA National Championships. This is comparable to the peak age for athletes at the 90^th^ percentile at IWF World championships, although heavier athletes may have a higher peak age [16,27]. In comparison, in throwing disciplines in track and field world championships, men reached their peak at an older age, 28.0 ± 0.4 years [14], corresponding to a higher peak age in weightlifters with higher body mass. Athletes in jumping disciplines reached peak performances at 25.8 ± 0.3 years for men and 25.6 ± 0.4 years for women [14], which corresponded to the overall peak age for weightlifters. On the other hand, weightlifters with a performance level at the 50^th^ percentile reached peak performance at slightly older ages compared to those at the 90^th^ percentile, namely 27.8 years (CI: 27.1, 28.5) for men and 27.6 years (CI: 26.8, 28.4) years for women.

### Limitations

There are a number of limitations to this study. First, the database does not contain sufficient data for a longitudinal analysis of athletes. Less than one percent of the athletes in the senior age category also competed as juniors or youth. The participation is sparser at National Senior championships, possibly due to higher qualifying standards or because of socioeconomic reasons. This could account for the brief plateau in sex difference in the transition to from junior to senior age groups. Second, anthropometric and training variables were not considered. The biological age and training age are more closely associated with performance than the chronological age used in this study. However, body mass, as an indicator of the physique of the athlete, enabled us to compare performance levels for athletes of different body mass. Third, while undetected doping violations cannot be excluded from the data in our study, we excluded all results from athletes who were found to violate anti-doping policies at any one time point.

## Conclusion

We quantified the impact of body weight, age, and sex differences for youth and young adults, ages 6 to 30 years old, participating in national level Olympic weightlifting competitions in the United States. Body weight at younger ages has less impact on performance compared to older ages, and boys and girls perform similarly. When reaching the ages typically associated with the onset of puberty, boys’ performances rapidly increase and the gap between genders widens. Women achieve peak performance at a similar or slightly younger age than men. Such results may help to establish progression trajectories for young athletes and may aid key stakeholders in evaluating expected improvements taking into account sex-specific differences.
